# Inhibitors of apoptosis: expression and regulation in the endometrium during the estrous cycle and at the maternal-conceptus interface during pregnancy in pigs

**DOI:** 10.5713/ab.21.0307

**Published:** 2021-09-15

**Authors:** Inkyu Yoo, Wonchul Jung, Soohyung Lee, Yugyeong Cheon, Hakhyun Ka

**Affiliations:** 1 Department of Biological Science and Technology, Yonsei University, Wonju 26493, Korea

**Keywords:** Apoptosis, Caspase, Endometrium, Inhibitors of Apoptosis, Pig

## Abstract

**Objective:**

Caspase-mediated apoptosis plays a crucial role in the regulation of endometrial and placental function in females. Caspase activity is tightly controlled by members of the inhibitors of apoptosis proteins (IAPs) family. However, the expression and regulation of IAPs at the maternal-conceptus interface has not been studied in pigs. Therefore, we determined the expression of IAP family members baculovirus IAP repeat-containing 1 (*BIRC1*) to *BIRC6* at the maternal-conceptus interface in pigs.

**Methods:**

We obtained endometrial tissues from pigs at various stages of the estrous cycle and pregnancy, conceptus tissues during early pregnancy, and chorioallantoic tissues during mid- to late pregnancy and analyzed the expression of IAPs. Furthermore, we determined the effects of the steroid hormones estradiol-17β (E2) and progesterone on the expression of IAPs in endometrial explant tissue cultures.

**Results:**

During the estrous cycle, *BIRC2* and *BIRC5* expression varied cyclically, and during pregnancy, endometrial *BIRC1*, *BIRC2*, *BIRC3*, *BIRC4*, and *BIRC5* expression varied in a stage-specific manner. Conceptus and chorioallantoic tissues also expressed IAPs during pregnancy. The *BIRC2* and *BIR3* mRNAs were localized to luminal epithelial cells, and BIRC4 proteins to glandular epithelial cells in the endometrium. Exposure of endometrial tissues to E2 increased the expression of *BIRC6*, while progesterone increased the expression of *BIRC1*, *BIRC4*, and *BIRC6* in a dose-dependent manner.

**Conclusion:**

These results indicated that IAPs were expressed in the endometrium during the estrous cycle and at the maternal-conceptus interface during pregnancy in a stage-specific manner. In addition, steroid hormones were found to be responsible for the expression of some IAPs in pigs. Together, the results suggested that IAPs may play important roles in endometrial and placental functions by regulating caspase action and apoptosis at the maternal-conceptus interface.

## INTRODUCTION

Apoptosis, a process of programmed cell death, is an important cellular event in many biological processes, including tissue homeostasis, development, and immunity [1]. Apoptosis also plays a critical role in reproductive processes such as functional maintenance of the endometrium and embryo implantation [2]. In humans, apoptosis allows the elimination of senescent cells from the functional layer of the endometrium during the menstrual cycle [2]. During early pregnancy, interactions between the embryo and endometrium facilitate adhesion and invasion of the implanting embryo, which leads to complete apoptosis of the epithelial layer at the implantation site [3]. In non-primate species, menstruation does not occur as part of the estrous cycle, but apoptosis is nonetheless involved in endometrial remodeling during cyclical cell proliferation and degeneration. In mice, apoptosis in the uterine tissue is regulated by ovarian steroid hormones during the estrous cycle and occurs in the decidua after implantation [4–6] In pigs, apoptotic cells have been detected in the endometrium during the estrous cycle and pregnancy, mostly in the stroma at the time of implantation [7,8]. Our previous study in pigs showed that many maternal T cells are recruited into the endometrium during the implantation period [9], and some T cells expressing cell death receptors undergo apoptosis [8].

Apoptotic cell death is mediated by a family of cysteine-aspartic proteases called caspases (CASPs) that are widely expressed in most cells [1]. The CASPs associated with apoptosis are classified as initiator (CASP2, CASP8, CASP9, and CASP10) or executioner (CASP3, CASP6, and CASP7) CASPs according to their activity [10]. Initiator CASPs activate other executioner CASPs [11,12]. Executioner CASPs, especially CASP3 and CASP7, cleave various substrates that are essential for cell survival. The cleavage of cell survival factors results in degradation of cytoskeletal proteins and DNA fragmentation, leading to cell death without spillage of its contents [1, 11,13]. Our recent study in pigs indicated that the expression of *CASP3* and *CASP7* increases in the endometrium at the time of implantation, and an active form of CASP3 is localized to endometrial epithelial and stromal cells [14]. Interestingly, during early pregnancy, we detected only a few apoptotic cells in the endometrial stroma and none in the epithelium [14], suggesting a mechanism to regulate apoptosis in a cell-type specific manner may be present in the endometrium during the implantation period in pigs.

Caspase activity can be inhibited by members of the inhibitors of apoptosis proteins (IAPs) family, also known as the baculovirus IAP repeat-containing (BIRC) family [15,16]. To date, eight mammalian IAPs have been identified: neuronal apoptosis inhibitory protein (NAIP; BIRC1), cellular IAP1 (cIAP1; BIRC2), cIAP2 (BIRC3), X-linked inhibitor of apoptosis (XIAP; BIRC4), survivin (BIRC5), BIR-repeat-containing ubiquitin-conjugating enzyme (BRUCE; BIRC6), livin (BIRC7), and testis-specific IAP (Ts-IAP; BIRC8) [15,17]. The IAPs regulate ubiquitin-dependent signaling pathways including nuclear factor-κB and mitogen-activated protein kinase pathways that mediate inflammation, immune responses, cell migration and survival [18]. Among the various IAP members, BIRC2, BIRC3, and BIRC4 have been the most extensively studied in mammalian species. These three BIRCs each contain a carboxyl-terminal interesting new gene domain that provides E3 ubiquitin ligase activity and enables their degradation [19]. BIRC2 binds strongly to and inhibits CASP3 and CASP7 but not CASP9, and BIRC3 selectively binds to and inhibits CASP9 but not CASP3 or CASP7 [20,21], whereas BIRC4 is considered the most potent inhibitor of CASP3, CASP7, and CASP9 with nanomolar affinity [22].

A few studies have reported the expression and function of some IAPs in the endometrium and placentas in humans and rodents. In humans, BIRC3 and BIRC5 are expressed in the endometrium, and aberrant expression of these IAPs is associated to endometriosis and endometrial cancer [23–25]. In human placentas, IAPs are expressed primarily in cytotrophoblast cells and the syncytiotrophoblast layer during pregnancy, and BIRC3 expression prevents apoptosis in cytotrophoblast cells [26,27]. In addition, survival and proliferation of trophoblast cells are also related to the expression of BIRC4 and BIRC5, respectively, during pregnancy in humans and rodents [28–30]. Although the expression and function of IAPs in the endometrium and placenta have been investigated in humans and rodents, little is known about the expression, regulation, and function of IAPs in the endometrium during the estrous cycle and pregnancy in pigs.

Because CASP3 and CASP7 are expressed in the endometrial epithelium at the time of conceptus implantation without causing apoptotic cell death, and IAPs are responsible for inhibiting caspases, we postulated that IAPs may regulate endometrial epithelial cell differentiation and homeostasis during both the estrous cycle and pregnancy in pigs. Therefore, to initiate our study of the regulatory mechanism of apoptosis in the endometrium at the maternal-conceptus interface in pigs, we assessed i) the expression of IAPs for which porcine sequence information is available (*BIRC1*, *BIRC2*, *BIRC3*, *BIRC4*, *BIRC5*, and *BIRC6*) in the endometrium during the estrous cycle and pregnancy, in conceptus tissues during early pregnancy, and in chorioallantoic tissues during mid- to late pregnancy; ii) the localization of IAPs in the endometrium; and iii) the regulation of endometrial tissue IAP expression by the steroid hormones estrogen and progesterone.

## MATERIALS AND METHODS

### Animals and tissue preparation

All experimental procedures involving animals were conducted in accordance with the Guide for the Care and Use of Research Animals in Teaching and Research and approved by the Institutional Animal Care and Use Committee of Yonsei University (No. YWC-P120) and the National Institute of Animal Science (No. 2015-137). Sexually mature Landrace-Yorkshire gilts of similar age (6 to 8 months) and weight (100 to 120 kg) were assigned randomly to either the cycling or pregnant groups, as described previously [31]. Gilts assigned to the pregnant group were artificially inseminated with fresh boar semen at the onset of estrus (day 0) and 12 h later. The reproductive tracts of the gilts were obtained immediately after slaughter on days 0, 3, 6, 9, 12, 15, or 18 of the estrous cycle or days 10, 12, 15, 30, 60, 90, or 114 of pregnancy (n = 3–6/d/status). Pregnancy was confirmed by the presence of apparently normal filamentous conceptuses in uterine flushings on days 10, 12, and 15 and the presence of embryos and placenta on later days of pregnancy. Conceptus tissues were obtained by uterine flushing on days 12 and 15 of pregnancy. Uterine flushing was performed by introducing and recovering 25 mL of phosphate-buffered saline (PBS; pH 7.4) into each uterine horn. Chorioallantoic tissues were obtained on days 30, 60, 90, and 114 of pregnancy (n = 3–4/d). Endometrial tissues from prepubertal gilts (n = 8; approximately 6 months of age) that had not undergone an estrous cycle and with no corpus luteum formed were obtained from a local slaughterhouse. Endometrium, dissected free of myometrium, was collected from the middle portion of each uterine horn, snap-frozen in liquid nitrogen, and stored at −80°C prior to RNA extraction. For immunohistochemistry, cross-sections of the endometrium were fixed in 4% paraformaldehyde in PBS (pH 7.4) for 24 h and then embedded in paraffin as previously described [32].

### Explant cultures

To determine the effects of the steroid hormones estradiol-17β (E2) and progesterone (P4) on the expression of six IAP mRNAs in the endometrium, endometrial explant tissues obtained from prepubertal gilts were cultured as previously described [14,31]. The endometrium was dissected free of the myometrium and placed into warm phenol-red-free Dulbecco’s modified Eagle’s medium/F-12 culture medium (DMEM/F-12; Sigma, St. Louis, MO, USA) containing penicillin G (100 IU/mL) and streptomycin (0.1 mg/mL). The endometrium was minced into small pieces (2 to 3 mm^3^) using scalpel blades, and 500 mg aliquots were placed into T25 flasks with serum-free modified DMEM/F-12 containing 10 μg/mL insulin (Sigma, USA), 10 ng/mL transferrin (Sigma, USA), and 10 ng/mL hydrocortisone (Sigma, USA). Endometrial explants were cultured immediately after mincing in the presence of increasing concentrations of E2 (0, 5, 50, or 500 pg/mL; Sigma, USA) or P4 (0, 0.3, 3, or 30 ng/mL; Sigma, USA) for 24 h with rocking in an atmosphere of 5% CO_2_ in air at 37°C. The selected concentrations encompassed the full range of physiological concentrations of E2 and P4 [33]. Explant tissues were then harvested, and total RNA was extracted for real-time reverse transcription-polymerase chain reaction (RT-PCR) to determine the expression of *BIRC1*, *BIRC2*, *BIRC3*, *BIRC4*, *BIRC5*, and *BIRC6* mRNAs.

### Total RNA extraction, reverse transcription-polymerase chain reaction, and cloning of six porcine IAP cDNAs

Total RNA was extracted from endometrial, conceptus, and chorioallantoic tissues using TRIzol reagent (Invitrogen, Carlsbad, CA, USA) according to the manufacturer’s recommendations. The quantity of RNA was assessed spectrophotometrically, and RNA integrity was validated following electrophoresis in 1% agarose gel. Four micrograms of total RNA were treated with DNase I (Promega, Madison, WI, USA) and reverse transcribed using SuperScript II Reverse Transcriptase (Invitrogen, USA) to obtain complementary DNAs (cDNAs). The cDNA templates were then diluted 1:4 with nuclease-free water and amplified by PCR using Taq polymerase (Takara Bio, Shiga, Japan) and specific primers based on porcine IAP mRNA sequences. The PCR conditions, sequences of primer pairs for IAPs and expected product sizes are listed in Table 1. The PCR products were separated on 2% agarose gels and visualized using ethidium bromide staining. The identity of each amplified PCR product was verified by sequence analysis after cloning into the TOPOII vector (Invitrogen, USA).

### Quantitative real-time reverse transcription-polymerase chain reaction

To analyze the expression of six IAP mRNAs in endometrial and chorioallantoic tissues, real-time RT-PCR was performed using the Applied Biosystems StepOnePlus Real-Time PCR System (Applied Biosystems, Foster City, CA, USA) and the SYBR Green method. Complementary DNAs were synthesized from 4 μg total RNA isolated from each endometrial tissue, and newly synthesized cDNAs (total volume of 21 μL) were diluted 1:4 with nuclease-free water and then used for PCR. The Power SYBR Green PCR Master Mix (Applied Biosystems, USA) was used for PCR reactions. The final reaction volume of 20 μL included 2 μL of cDNA, 10 μL of 2X Master mix, 2 μL of each primer (100 nM), and 4 μL of dH_2_O. The PCR conditions and sequences of primer pairs are listed in Table 1. The results were reported as expression relative to that detected on day 0 of the estrous cycle, that on day 30 of pregnancy in chorioallantoic tissues, or that in control explant tissues after normalization of the amount of transcript to the geometric mean of endogenous porcine ribosomal protein L7 (*RPL7*) and ubiquitin B (*UBB*), and TATA binding protein (*TBP*) controls, all using the 2^−ΔΔCT^ method as previously described [34].

### *In situ* hybridization

The nonradioactive *in situ* hybridization procedure was performed as described previously [35], with some modifications. Sections (5 μm thick) were rehydrated by soaking them in successive xylene, 100% ethanol, 95% ethanol, and diethylpyrocarbonate (DEPC)-treated water baths. Tissue sections were boiled in citrate buffer (pH 6.0) for 10 min. After washing in DEPC-treated PBS, sections were digested using 5 μg/mL proteinase K (Sigma, USA) in 100 mM Tris-HCl and 50 mM ethylenediaminetetraacetic acid, pH 7.5, at 37°C. After post-fixation in 4% paraformaldehyde, tissue sections were incubated twice in PBS containing 0.1% active DEPC for 15 min and equilibrated for 15 min in 5× saline sodium citrate (SSC). The sections were prehybridized for 2 h at 68°C in hybridization mix (50% formamide, 5× SSC, 500 μg/mL herring sperm DNA, and 250 μg/mL yeast tRNA). Sense and antisense *BIRC2* and *BIRC3* riboprobes labeled with digoxigenin (DIG)-UTP were denatured for 5 min at 80°C and added to the hybridization mix. The hybridization reaction was carried out overnight at 68°C. Prehybridization and hybridization reactions were performed in a box saturated with a 5× SSC and 50% formamide solution to avoid evaporation, and no coverslips were used. After hybridization, sections were washed for 30 min in 2× SSC at room temperature, 1 h in 2× SSC at 65°C, and 1 h in 0.1× SSC at 65°C. Probes bound to the sections were detected immunologically using sheep anti-DIG Fab fragments covalently coupled to alkaline phosphatase and nitro blue tetrazolium chloride/5-bromo-4-chloro-3-indolyl phosphate (toluidine salt) as chromogenic substrate, according to the manufacturer’s protocol (Roche, Indianapolis, IN, USA).

### Immunohistochemistry

To identify the type(s) of porcine endometrial cells expressing BIRC4, sections were immunostained. Sections (5 μm thick) from paraffin-embedded tissues were deparaffinized and rehydrated in an alcohol gradient. Tissue sections were boiled in citrate buffer, pH 6.0, for 10 min, then washed with PBS and blocked with 0.5% (v/v) H_2_O_2_ in methanol for 30 min. Tissue sections were then blocked with 10% normal goat serum for 30 min at room temperature. Rabbit polyclonal anti-BIRC4 antibody (3 μg/mL; OriGene Technologies, Rockville, MD, USA) was added, and sections were incubated overnight at 4°C in a humidified chamber. For each tissue tested, purified normal rabbit immunoglobulin G was substituted for the primary antibody as a negative control. Tissue sections were washed three times with PBST. Biotinylated goat anti-rabbit secondary antibody (1 μg/mL; Vector Laboratories, Burlingame, CA, USA) was added, and sections were incubated for 1 h at room temperature. Following washes with PBST, a streptavidin-peroxidase conjugate (Invitrogen, USA) was added to the tissue sections, which were then incubated for 10 min at room temperature. The sections were washed with PBST, and a Peroxidase Substrate Kit (Vector Laboratories, USA) mixture was added to the tissue sections, which were then incubated for 10 min at room temperature. The tissue sections were washed in water, counterstained with Mayer’s hematoxylin, and coverslipped.

### Statistical analyses

Real-time RT-PCR data for six IAPs were subjected to an analysis of variance using the general linear models of SAS (Cary, NC, USA). As sources of variation, models included day, pregnancy status (cyclic or pregnant, days 12 and 15 post-estrus), and interactions to evaluate steady-state expression of the selected IAP mRNAs. Real-time RT-PCR data were analyzed by least squares regression analyses to assess the effects of day of the estrous cycle (day 0, 3, 6, 9, 12, 15, and 18) and pregnancy (day 10, 12, 15, 30, 60, 90, and 114) in the endometrium, and the effects of day of pregnancy in chorioallantoic tissue (day 30, 60, 90, and 114), and the effect of E2 and P4 on IAP expression in endometrial explant tissues. Data are presented as means with the standard errors of the mean. A p-value less than 0.05 was considered significant, whereas p-values if 0.05 to 0.10 were considered indicative of a trend toward significance.

## RESULTS

### Expression of BIRC1, BIRC2, BIRC3, BIRC4, BIRC5, and BIRC6 mRNAs in the endometrium during the estrous cycle and pregnancy in pigs

**Figure 1:**
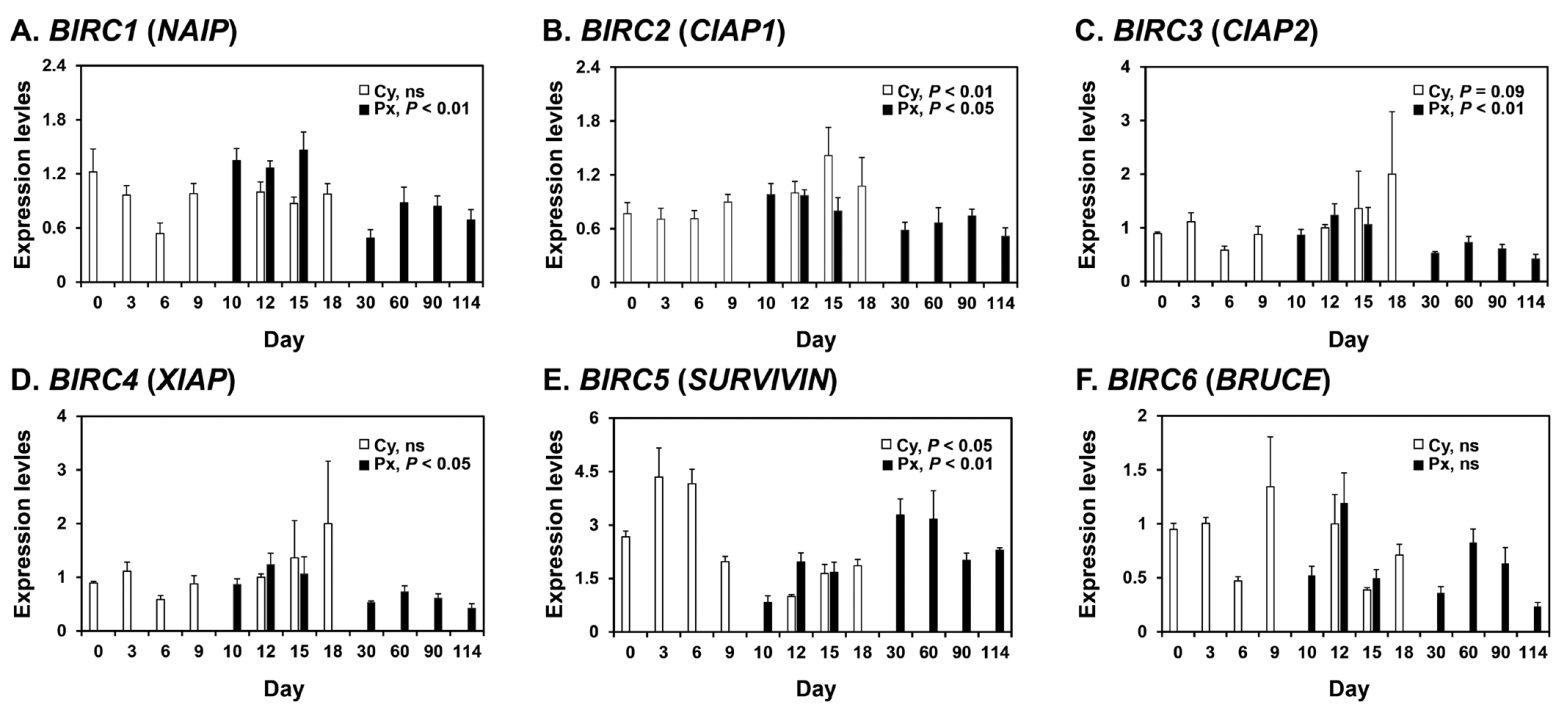
Expression of *BIRC1* (A), *BIRC2* (B), *BIRC3* (C), *BIRC4* (D), *BIRC5* (E) and *BIRC6* (E) mRNAs in the endometrium during the estrous cycle and pregnancy in pigs. Endometrial tissue samples from cyclic (Cy) and pregnant (Px) gilts were analyzed by real-time RT-PCR, and data are reported as the expression relative to that detected on day 0 of the estrous cycle after normalization to the transcript amount of the endogenous *RPL7*, *TBP*, and *UBB* mRNAs. Data are presented as the mean with standard error. *BIRC1*, baculovirus IAP repeat-containing 1; real-time RT-PCR, reverse transcription-polymerase chain reaction; *RPL7*, ribosomal protein L7; *TBP*, TATA binding protein; *UBB*, ubiquitin B. Statistical significances for the effect of day during the estrous cycle and pregnancy are indicated; ns, not significant.

To determine whether mRNAs for the *BIRC1*, *BIRC2*, *BIRC3*, *BIRC4*, *BIRC5*, and *BIRC6* IAPs were expressed in the endometrium of pigs, we performed real-time RT-PCR analyses (Figure 1). During the estrous cycle, the expression of *BIRC2* and *BIRC5*, but not other IAPs, varied cyclically, with increased expression of *BIRC2* at proestrus (linear effect of day, p<0.01) and *BIRC5* at metestrus (linear effect of day, p<0.05). On days 12 and 15 post-estrus, BIRC5 expression was affected by pregnancy status (p<0.05), but not by day or day×pregnancy status, and the expression of *BIRC5* was greater in the endometrium on day 12 of pregnancy than during the estrous cycle (p<0.05). The expression of *BIRC4* and *BIRC6* was affected by day (p<0.05), but not by pregnancy status or day×pregnancy status, with greater expression on day 12 than day 15. The steady-state expression of *BIRC1*, *BIRC2*, *BIRC3*, *BIRC4*, and *BIRC5*, but not *BIRC6*, was altered during pregnancy, with increased expression of *BIRC1*, *BIRC2*, *BIRC3*, and *BIRC4* during early pregnancy (linear effect of day, p<0.01 for *BIRC1* and *BIRC3*; p<0.05 for *BIRC2* and *BIRC4*) and increased expression of *BIRC5* during mid- to late pregnancy (cubic effect of day, p<0.01).

### Expression of BIRC1, BIRC2, BIRC3, BIRC4, BIRC5, and BIRC6 mRNAs in conceptuses during early pregnancy and chorioallantoic tissue during later stages of pregnancy

**Figure 2:**
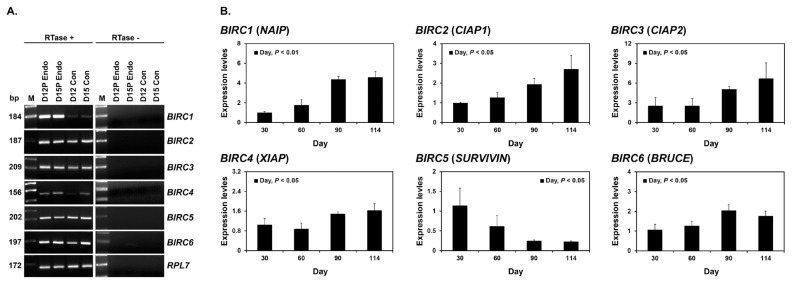
Expression of IAPs by conceptuses from days 12 and 15 of pregnancy (A) and by chorioallantoic tissues from mid- to late pregnancy (B). (A) RT-PCR analyses of *BIRC1–6* mRNAs from pregnancy days 12 and 15 conceptuses using total RNA. *RPL7* was used as a positive control. RTase +/-, with (+) or without (-) reverse transcriptase; M, molecular marker; D12P Endo, endometrium on day 12 of pregnancy; D15P Endo, endometrium on day 15 of pregnancy; D12 Con, day 12 conceptus; D15 Con, day 15 conceptus. (B) Real-time RT-PCR analyses of the expression of *BIRC1–6* mRNAs in chorioallantoic tissues on days 30, 60, 90, and 114 of pregnancy. Data are reported as expression relative to that detected on day 30 of pregnancy after normalization to the transcript amount of the endogenous *RPL7*, *TBP*, and *UBB* mRNAs control, and data are presented as means with standard errors. IAPs, inhibitors of apoptosis proteins; RT-PCR, reverse transcription-polymerase chain reaction; *BIRC1*, baculovirus IAP repeat-containing 1; *RPL7*, ribosomal protein L7; *TBP*, TATA binding protein; *UBB*, ubiquitin B. Statistical significances for the effect of day during pregnancy are indicated; ns, not significant.

To determine whether *BIRC1*, *BIRC2*, *BIRC3*, *BIRC4*, *BIRC5*, and *BIRC6* were expressed by conceptuses during early pregnancy (days 12 and 15), we performed RT-PCR using cDNA from conceptuses from days 12 and 15 of pregnancy. All IAP mRNAs were detected in conceptus tissues at both day 12 and 15 of pregnancy (Figure 2A). To determine whether the expression of *BIRC1*, *BIRC2*, *BIRC3*, *BIRC4*, *BIRC5*, and *BIRC6* changed in chorioallantoic tissues during mid- to late pregnancy (from day 30 to the end of gestation), we performed real-time RT-PCR analyses. The expression of *BIRC1*, *BIRC2*, *BIRC3*, *BIRC4*, and *BIRC6* increased towards the end of pregnancy, whereas the expression of *BIRC5* decreased (linear effect of day, p<0.01 for *BIRC1* and *BIRC2*; p<0.05 for *BIRC3*, *BIRC4*, *BIRC5*, and *BIRC6*; Figure 2B).

### Localization of BIRC2 and BIRC3 mRNAs and BIRC4 protein in the endometrium on days 12 and 15 of pregnancy in pigs

**Figure 3:**
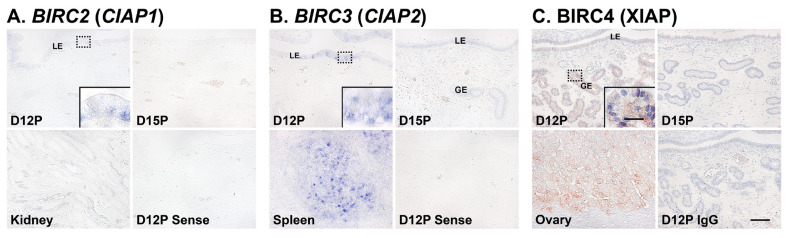
Localization of *BIRC2* (A) and *BIRC3* (B) mRNAs by *in situ* hybridization and BIRC4 (C) proteins by immunohistochemistry in the endometrium on days 12 and 15 of pregnancy in pigs. Representative uterine sections from day 12 of pregnancy stained with sense RNA probes or normal IgG (IgG) are shown as negative controls. Tissue sections from the kidney, spleen and ovary are shown as positive controls for *BIRC2* and *BIRC3* mRNAs and BIRC4 protein, respectively. *BIRC1*, baculovirus IAP repeat-containing 1; IgG, immunoglobulin G; D, day; C, estrous cycle; P, pregnancy; LE, luminal epithelium; GE, glandular epithelium. Bars = 100 μm and 20 μm in inset.

Next, we performed *in situ* hybridization analyses to determine which cell type(s) express *BIRC2* and *BIRC3* mRNAs in the endometrium on days 12 and 15 of pregnancy. The expression of *BIRC2* and *BIRC3* mRNAs was localized primarily to endometrial epithelial cells with strong signal intensity in luminal epithelial (LE) cells. We also performed immunohistochemistry to identify BIRC4 protein in the endometrium and found that BIRC4 protein was localized to glandular epithelial (GE) cells in the endometrium with strong signal intensity on day 12 of pregnancy (Figure 3C).

### Effects of steroid hormones E2 and P4 on BIRC1, BIRC2, BIRC3, BIRC4, BIRC5, and BIRC6 expression in endometrial tissues

**Figure 4:**
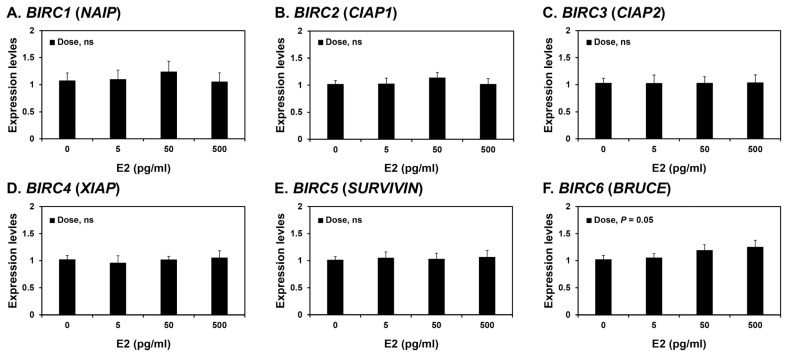
Effects of estradiol-17β (E2) on the expression of *BIRC1* (A), *BIRC2* (B), *BIRC3* (C), *BIRC4* (D), *BIRC5* (E), and *BIRC6* (E) mRNAs in endometrial explant cultures. Endometrial explants from prepubertal gilts were cultured with 0, 5, 50, or 500 pg/mL E2. Abundance of mRNA expression determined by real-time RT-PCR analyses is presented relative to that for *BIRC1–6* mRNAs in the control group (0 pg/mL E2) of endometrial explants after normalization of transcript amounts to *RPL7*, *TBP*, and *UBB* mRNAs. Data are presented as the means with standard errors. *BIRC1*, baculovirus IAP repeat-containing 1; real-time RT-PCR, reverse transcription-polymerase chain reaction; *RPL7*, ribosomal protein L7; *TBP*, TATA binding protein; *UBB*, ubiquitin B. Statistical significances for the effect of dose are indicated; ns, not significant. These treatments were performed in triplicate using tissues obtained from each of three gilts.

**Figure 5:**
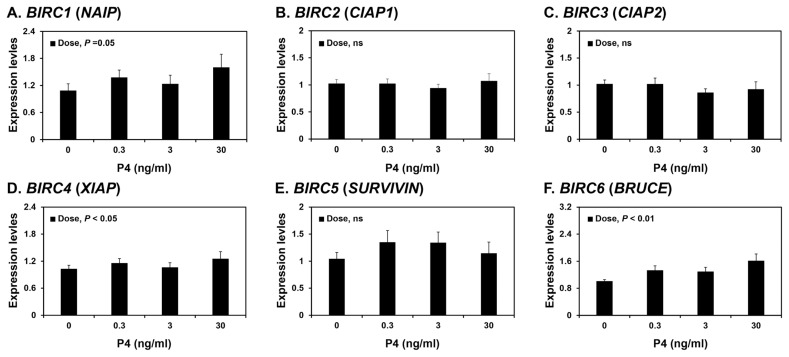
Effects of progesterone (P4) on the expression of *BIRC1* (A), *BIRC2* (B), *BIRC3* (C), *BIRC4* (D), *BIRC5* (E), and *BIRC6* (F) mRNAs in endometrial explant cultures. Endometrial explants from prepubertal gilts were cultured with 0, 0.3, 3, or 30 ng/mL P4. The mRNA expression as determined by real-time RT-PCR analyses is expressed relative to that for *BIRC1–6* mRNAs in the control group (0 ng/mL P4) of endometrial explants after normalization of transcript amounts to *RPL7*, *TBP*, and *UBB* mRNAs. Data are presented as means with standard errors. *BIRC1*, baculovirus IAP repeat-containing 1; real-time RT-PCR, reverse transcription-polymerase chain reaction; *RPL7*, ribosomal protein L7; *TBP*, TATA binding protein; *UBB*, ubiquitin B. Statistical significances for the effect of dose are indicated; ns, not significant. These treatments were performed in triplicate using tissues obtained from each of three gilts.

Because the expression of some *BIRCs* varied during the estrous cycle, we investigated whether steroid hormones, E2 and P4, affected the expression of IAPs, *BIRC1*, *BIRC2*, *BIRC3*, *BIRC4*, *BIRC5*, and *BIRC6*, in endometrial tissues. To assess the individual effects of E2 or P4, and to exclude the possibility that these hormones influenced endometrial tissues during the estrous cycle, we utilized endometrial tissues from prepubertal gilts that had not yet undergone an estrous cycle. When we cultured endometrial explant tissues with increasing concentrations of E2 or P4, P4 induced the expression of *BIRC1*, *BIRC4*, and *BIRC6* mRNAs (linear effect of dose, p<0.01; Figure 4), while E2 did not affect the expression of IAP expression in endometrial tissues (Figure 5).

## DISCUSSION

The novel findings of this study of female pigs were i) the endometrium expressed IAPs during the estrous cycle and pregnancy in a stage- and pregnancy-status-specific manner; ii) conceptuses on days 12 and 15 of pregnancy expressed IAPs; iii) chorioallantoic tissues from day 30 to the end of gestation had progressively increasing (*BIRC1*, *BIRC2*, *BIRC3*, *BIRC4*, and *BIRC6*) or decreasing (*BIRC5*) expression of IAPs; iv) *BIRC2* and *BIRC3* mRNAs were localized predominantly within endometrial LE cells and BIRC4 protein within endometrial GE cells during early pregnancy; v) estrogen induced the expression of *BIRC6*, and progesterone induced the expression of *BIRC1*, *BIRC4*, and *BIRC6* in a concentration-dependent manner in endometrial explant tissues.

The IAPs are important regulators of apoptosis and play crucial roles in a variety of biological processes, such as tissue homeostasis, development, and immunity [1]. Endometrial and placental IAP expression has been confirmed in both humans and rodents. In humans, BIRC4 is expressed in the endometrium and placenta during the reproductive cycle and pregnancy and is involved in regulation of endometrial remodeling and placental development [36]. Increased expression of BIRC2, BIRC3, BIRC4, and BIRC5 is associated with endometriosis, and tumor necrosis factor-α increases BIRC3 expression in endometrial stromal cells *in vitro* [24]. In addition, IAPs are expressed in cytotrophoblast cells and the syncytiotrophoblast layer during pregnancy [26]. However, the expression of IAP family members in the endometrium throughout the estrous cycle and at the maternal-conceptus interface during pregnancy has not been fully determined in any species. The present study demonstrated the dynamic expression of IAPs in the endometrium throughout the estrous cycle and pregnancy and in conceptus and chorioallantoic tissues during pregnancy in pigs.

The expression of *BIRC2*, *BIRC3*, and *BIRC5* varied cyclically during the estrous cycle, with peak *BIRC2* and *BIRC3* expression occurring during the proestrus phase and of *BIRC5* during the metestrus phase. Our previous study showed that caspases are expressed in the endometrium during the estrous cycle and pregnancy in pigs [14]. The expression pattern of *BIRC2* and *BIRC3* is similar to those of *CASP6*, *CASP7*, and *CASP8*, and the expression pattern of *BIRC5* is similar to *CASP10* during the estrous cycle [14]. Because BIRC2 and BIRC3 inhibit the action of CASP8 [37], and BIRC5 inhibits active forms of CASP3 and CASP7 [38], the results of this study suggest that differential expression of *BIRC2*, *BIRC3*, and *BIRC5* during the different stages of the estrous cycle is responsible for tight regulation of CASP3, CASP7, and CASP8 actions in the endometrium during the estrous cycle in pigs.

Although the expression and activity of IAPs have been demonstrated at the maternal-fetal interface of both humans and rodents [28,29,36,39], most of these studies have focused on the expression of BIRC4 and BIRC5. In this study, we found that all IAPs studied were expressed in the endometrium and their expression (except for BIRC6) varied in a stage-specific manner during pregnancy. The expression of *BIRC1*, *BIRC2*, *BIRC3*, and *BIRC4* was greater during early pregnancy than mid- to late pregnancy, suggesting that the expression of IAPs is pregnancy stage-specific and may play an important role in regulating endometrial functions for the establishment of pregnancy during the peri-implantation period. Expression of *BIRC5* was greater during mid-pregnancy than early or late pregnancy, a pattern that corresponded with the onset and completion of placentation. Thus, it is likely that increased *BIRC5* expression during mid-pregnancy may be involved in support of placental development. In addition, these results suggested that depending on the stage of pregnancy, different IAP members are responsible for inhibition of apoptosis in the endometrium.

We also determined the cell type(s) expressing IAPs mRNAs or proteins in the endometrium during the estrous cycle and early pregnancy. Our results showed that *BIRC2* and *BIRC3* mRNAs were localized to LE cells and BIRC4 proteins to GE cells during early pregnancy. Our previous study demonstrated that active CASP3 proteins are localized to LE and stromal cells and CASP7 is localized to LE cells during early pregnancy, but cells undergoing apoptosis in the endometrium were detected only in the stroma [14]. Notably, the endometrial expression of *CASP3* and *CASP7* is induced by conceptus signals, interleukin-1β and interferon-γ, respectively [14]. The executioner caspases CASP3, CASP6, and CASP7 play important roles not only in apoptotic cell death, but also in cell differentiation in various cell types, including muscle cells, neurons, stem cells, trophoblasts, and epithelial cells [40]. Indeed, endometrial epithelial cells undergo a dramatic differentiation process during the implantation period to establish cell-to-cell interactions with trophectodermal cells to form a true epitheliochorial type of placenta in pigs [41]. The process of endometrial LE cell differentiation during the implantation period comprises altered epithelial morphology, increased secretory activity, and increased expression of immunity-related molecules in response to conceptus signals, estrogen, interleukin-1β, or interferon-γ [14,33]. Thus, it is likely that CASP3 and CASP7 are involved in the differentiation process of endometrial LE cells, and IAPs expressed in LE cells regulate CASP3 and CASP7 activity to inhibit caspase-mediated apoptotic cell death and protect LE cells. However, the detailed mechanisms of action of caspases and IAPs in endometrial LE cells during the differentiation process still need to be clarified.

The expression of CASP6, CASP7, CASP8, CASP9, and CASP10 in chorioallantoic tissues increases from mid-pregnancy to the end of gestation, although apoptotic cells are barely detectable in chorioallantoic tissues during pregnancy in pigs [14]. Our study results showed that IAPs were expressed in conceptus tissues during early pregnancy and in chorioallantoic tissues during mid- to late pregnancy. In particular, the expression of *BIRC1*, *BIRC2*, *BIRC3*, *BIRC4*, and *BIRC6* in chorioallantoic tissues increased progressively from conception to the end of gestation. Expression of IAPs in cytotrophoblast cells and the syncytiotrophoblast layer in the human placentas protects cytotrophoblast cells from undergoing apoptosis [26,27]. Thus, our results suggest that the increased expression of IAPs in chorioallantoic tissues during mid- to late pregnancy is responsible for regulation of caspase activity to protect chorioallantoic tissues from undergoing apoptosis. Interestingly, *BIRC5* expression in chorioallantoic tissues decreased toward the end of the gestation period in this study. In abortion-prone mice, increased placental BIRC5 expression during early pregnancy is associated with pregnancy loss, although the role of BIRC5 in pregnancy failure is not fully understood [39]. Thus, tight regulation of BIRC5 expression in the placenta may be critical for the maintenance of successful pregnancy, but the specific mechanism of BIRC5 regulation of chorioallantoic tissues apoptosis and pregnancy outcomes needs further study.

In this study, endometrial expression of *BIRC2*, *BIRC3*, and *BIRC5* varied during the porcine estrous cycle, while expression of *BIRC1*, *BIRC2*, *BIRC3*, *BIRC4*, and *BIRC5* varied during pregnancy. Although transcriptional regulation of IAPs is not well understood, some studies have reported that the steroid hormones E2 and P4, together with the cytokines’ tumor necrosis factor-α and transforming growth factor-β, regulate IAP expression in the endometrium in humans and rats [24,42–44]. In humans, the expression of *BIRC5* in endometrial tissues is induced by E2 and reduced by P4 [44]; in contrast, endometrial *BIRC4* expression is induced by E2 in ovariectomized rats [42]. Thus, differential expression of IAPs in the endometrium led us to postulate that ovarian steroid hormones might be involved in regulation of endometrial IAP expression. Indeed, in this study, E2 increased the expression of *BIRC6* in porcine endometrial explant tissue cultures, and P4 increased the expression of *BIRC1*, *BIRC4*, and *BIRC6*, suggesting that the expression of some IAPs in the endometrium during the estrous cycle and pregnancy are regulated by steroid hormones. Although we have not determined the effect of tumor necrosis factor-α and transforming growth factor-β on IAP expression, it is possible that these and other factors may also affect the expression of IAPs in endometrial and chorioallantoic tissues in pigs.

In conclusion, the results of this study in pigs indicated that IAPs were expressed in the endometrium during the estrous cycle and pregnancy, and in conceptus and chorioallantoic tissues during pregnancy; *BIRC2* and *BIRC3* mRNAs were localized to endometrial LE cells, and BIRC4 proteins were localized to GE cells; and the steroid hormones E2 and/or P4 regulated the expression of some IAPs in endometrial tissues. These results suggest that stage-specific IAP expression in the endometrium during the estrous cycle and pregnancy and in chorioallantoic tissues during pregnancy may play important roles in reproductive cyclicity and in the establishment and maintenance of pregnancy by regulating apoptosis and epithelial differentiation in pigs.

## Figures and Tables

**Table 1:** Summary of primer sequences for RT-PCR, real-time RT-PCR, and *in situ* hybridization and expected product sizes

Primer	Sequence of forward (F) and reverse (R) primers (5′ → 3′)	Annealing temperature (°C)	Product size (bp)	GenBank accession no.
For RT-PCR and real-time RT-PCR				
* * *BIRC1*	F: TCCCCTTCTGGAAGTTGATG R: AATGGAGCACTGGGTGAAAG	60	184	XM_021076820.1
* * *BIRC2*	F: ACACATGTGGCTCGACTGAG R: ACCACTTGGCGTGTTCTACC	60	187	XM_021062708.1
* * *BIRC3*	F: GATGAAATAAGGGAAGAGGAGAAAG R: TCTCTTGCTTGTAAAGATGTCTGTG	60	209	XM_021062707.1
* * *BIRC4*	F: AATCCTGGTGTCCAAAATGG R: AGGGTTCCTCGGGTATATGG	60	156	EF120983.1
* * *BIRC5*	F: CCTGGCAGCTCTACCTCAAG R: TCTTCTATGGGGTCGTCGTC	60	202	NM_214141.1
* * *BIRC6*	F: AGCTCCAGGCTTAGGGAAAG R: GATCAAGGCCACTAGCAAGC	60	197	XM_021087656.1
* * *RPL7*	F: AAG CCA AGC ACT ATC ACA AGG AAT ACA R: TGC AAC ACC TTT CTG ACC TTT GG	60	172	NM_001113217
* * *TBP*	F: AACAGTTCAGTAGTTATGAGCCAGA R: AGATGTTCTCAAACGCTTCG	60	262	DQ845178.1
* * *UBB*	F: GCATTGTTGGCGGTTTCG R: AGACGCTGTGAAGCCAATCA	60	81	NM_001105309.1
For In situ hybridization				
* * *BIRC2*	F: ACACATGTGGCTCGACTGAG R: TAGGAAGCACGCATGTCAAC	60	631	XM_021062708.1
* * *BIRC3*	F: CCATTGTGCAATGAATACCG R: TCTCTTGCTTGTAAAGATGTCTGTG	60	997	XM_021062707.1

RT-PCR, reverse transcription-polymerase chain reaction; *BIRC1*, baculovirus IAP repeat-containing 1; *RPL7*, ribosomal protein L7; *TBP*, TATA binding protein; *UBB*, ubiquitin B.

## References

[1] Elmore S (2007). Apoptosis: a review of programmed cell death. Toxicol Pathol.

[2] Harada T, Kaponis A, Iwabe T (2004). Apoptosis in human endometrium and endometriosis. Hum Reprod Update.

[3] Cohen M, Wuillemin C, Irion O, Bischof P (2010). Role of decidua in trophoblastic invasion. Neuro Endocrinol Lett.

[4] Annie L, Gurusubramanian G, Roy VK (2019). Estrogen and progesterone dependent expression of visfatin/NAMPT regulates proliferation and apoptosis in mice uterus during estrous cycle. J Steroid Biochem Mol Biol.

[5] Pampfer S, Donnay I (1999). Apoptosis at the time of embryo implantation in mouse and rat. Cell Death Differ.

[6] Tan J, Raja S, Davis MK, Tawfik O, Dey SK, Das SK (2002). Evidence for coordinated interaction of cyclin D3 with p21 and cdk6 in directing the development of uterine stromal cell decidualization and polyploidy during implantation. Mech Dev.

[7] Okano A, Ogawa H, Takahashi H, Geshi M (2007). Apoptosis in the porcine uterine endometrium during the estrous cycle, early pregnancy and post partum. J Reprod Dev.

[8] Yoo I, Kye YC, Han J (2020). Uterine epithelial expression of the tumor necrosis factor superfamily: a strategy for immune privilege during pregnancy in a true epitheliochorial placentation species. Biol Reprod.

[9] Han J, Gu MJ, Yoo I (2017). Analysis of cysteine-X-cysteine motif chemokine ligands 9, 10, and 11, their receptor CXCR3, and their possible role on the recruitment of immune cells at the maternal-conceptus interface in pigs. Biol Reprod.

[10] McIlwain DR, Berger T, Mak TW (2013). Caspase functions in cell death and disease. Cold Spring Harb Perspect Biol.

[11] Green DR, Llambi F (2015). Cell death signaling. Cold Spring Harb Perspect Biol.

[12] Galluzzi L, Lopez-Soto A, Kumar S, Kroemer G (2016). Caspases connect cell-death signaling to organismal homeostasis. Immunity.

[13] D'Arcy MS (2019). Cell death: a review of the major forms of apoptosis, necrosis and autophagy. Cell Biol Int.

[14] Jung W, Yoo I, Han J (2021). Expression of caspases in the pig endometrium throughout the estrous cycle and at the maternal-conceptus interface during pregnancy and regulation by steroid hormones and cytokines. Front Vet Sci.

[15] Deveraux QL, Reed JC (1999). IAP family proteins—suppressors of apoptosis. Genes Dev.

[16] Kenneth NS, Duckett CS (2012). IAP proteins: regulators of cell migration and development. Curr Opin Cell Biol.

[17] Saleem M, Qadir MI, Perveen N (2013). Inhibitors of apoptotic proteins: new targets for anticancer therapy. Chem Biol Drug Des.

[18] Silke J, Meier P (2013). Inhibitor of apoptosis (IAP) proteins-modulators of cell death and inflammation. Cold Spring Harb Perspect Biol.

[19] Hu S, Yang X (2003). Cellular inhibitor of apoptosis 1 and 2 are ubiquitin ligases for the apoptosis inducer Smac/DIABLO. J Biol Chem.

[20] Eckelman BP, Salvesen GS, Scott FL (2006). Human inhibitor of apoptosis proteins: why XIAP is the black sheep of the family. EMBO Rep.

[21] Shiozaki EN, Shi Y (2004). Caspases, IAPs and Smac/DIABLO: mechanisms from structural biology. Trends Biochem Sci.

[22] Deveraux QL, Takahashi R, Salvesen GS, Reed JC (1997). X-linked IAP is a direct inhibitor of cell-death proteases. Nature.

[23] Watanabe A, Taniguchi F, Izawa M (2009). The role of survivin in the resistance of endometriotic stromal cells to drug-induced apoptosis. Hum Reprod.

[24] Taniguchi F, Higaki H, Izawa M (2014). The cellular inhibitor of apoptosis protein-2 is a possible target of novel treatment for endometriosis. Am J Reprod Immunol.

[25] Neubauer NL, Ward EC, Patel P (2011). Progesterone receptor-B induction of BIRC3 protects endometrial cancer cells from AP1-59-mediated apoptosis. Horm Cancer.

[26] Ka H, Hunt JS (2003). Temporal and spatial patterns of expression of inhibitors of apoptosis in human placentas. Am J Pathol.

[27] Gill RM, Hunt JS (2004). Soluble receptor (DcR3) and cellular inhibitor of apoptosis-2 (cIAP-2) protect human cytotrophoblast cells against LIGHT-mediated apoptosis. Am J Pathol.

[28] Shooner C, Caron PL, Frechette-Frigon G, Leblanc V, Déry MC, Asselin E (2005). TGF-beta expression during rat pregnancy and activity on decidual cell survival. Reprod Biol Endocrinol.

[29] Straszewski-Chavez SL, Abrahams VM, Aldo PB, Romero R, Mor G (2010). AKT controls human first trimester trophoblast cell sensitivity to FAS-mediated apoptosis by regulating XIAP expression. Biol Reprod.

[30] Fest S, Brachwitz N, Schumacher A (2008). Supporting the hypothesis of pregnancy as a tumor: survivin is upregulated in normal pregnant mice and participates in human trophoblast proliferation. Am J Reprod Immunol.

[31] Lee S, Yoo I, Han J, Ka H (2021). Antimicrobial peptides cathelicidin, PMAP23, and PMAP37: Expression in the endometrium throughout the estrous cycle and at the maternal-conceptus interface during pregnancy and regulation by steroid hormones and calcitriol in pigs. Theriogenology.

[32] Seo H, Kim M, Choi Y, Lee CK, Ka H (2008). Analysis of lysophosphatidic acid (LPA) receptor and LPA-induced endometrial prostaglandin-endoperoxide synthase 2 expression in the porcine uterus. Endocrinology.

[33] Ka H, Seo H, Choi Y, Yoo I, Han J (2018). Endometrial response to conceptus-derived estrogen and interleukin-1beta at the time of implantation in pigs. J Anim Sci Biotechnol.

[34] Livak KJ, Schmittgen TD (2001). Analysis of relative gene expression data using real-time quantitative PCR and the 2(-Delta Delta C(T)) method. Methods.

[35] Braissant O, Wahli W (1998). Differential expression of peroxisome proliferator-activated receptor-alpha, -beta, and -gamma during rat embryonic development. Endocrinology.

[36] Gruslin A, Qiu Q, Tsang BK (2001). X-linked inhibitor of apoptosis protein expression and the regulation of apoptosis during human placental development. Biol Reprod.

[37] Graber TE, Holcik M (2011). Distinct roles for the cellular inhibitors of apoptosis proteins 1 and 2. Cell Death Dis.

[38] Tamm I, Wang Y, Sausville E (1998). IAP-family protein survivin inhibits caspase activity and apoptosis induced by Fas (CD95), Bax, caspases, and anticancer drugs. Cancer Res.

[39] Garcia MG, Tirado-Gonzalez I, Handjiski B (2007). High expression of survivin and down-regulation of Stat-3 characterize the feto-maternal interface in failing murine pregnancies during the implantation period. Placenta.

[40] Shalini S, Dorstyn L, Dawar S, Kumar S (2015). Old, new and emerging functions of caspases. Cell Death Differ.

[41] Burghardt RC, Johnson GA, Jaeger LA (2002). Integrins and extracellular matrix proteins at the maternal-fetal interface in domestic animals. Cells Tissues Organs.

[42] Leblanc V, Dery MC, Shooner C, Asselin E (2003). Opposite regulation of XIAP and Smac/DIABLO in the rat endometrium in response to 17beta-estradiol at estrus. Reprod Biol Endocrinol.

[43] Caron PL, Frechette-Frigon G, Shooner C, Leblanc V, Asselin E (2009). Transforming growth factor beta isoforms regulation of Akt activity and XIAP levels in rat endometrium during estrous cycle, in a model of pseudopregnancy and in cultured decidual cells. Reprod Biol Endocrinol.

[44] Nabilsi NH, Broaddus RR, McCampbell AS (2010). Sex hormone regulation of survivin gene expression. J Endocrinol.

